# The role of statistics in advancing nitric oxide research in plant biology: from data analysis to mechanistic insights

**DOI:** 10.3389/fpls.2025.1597030

**Published:** 2025-07-01

**Authors:** Halah Fadhil Hussein AL-Hakeem, Murtaza Khan

**Affiliations:** ^1^ Post-Graduate Institute for Accounting & Financial Studies, University of Baghdad, Baghdad, Iraq; ^2^ Agriculture and Life Science Research Institute, Kangwon National University, Chuncheon, Republic of Korea

**Keywords:** nitric oxide, plant biology, statistical analysis, machine learning, systems biology

## Abstract

Nitric oxide (NO), a key signaling molecule in plants, induces various biological and biochemical processes, including growth and development, adaptive responses, and signaling pathways. The intricate nature of NO dynamics requires vigorous statistical approaches to guarantee precise data interpretation and significant biological conclusions. This review underscores the importance of statistical methodologies in NO study, discussing experimental design, data collection, and advanced analytical tools. In addition, vital statistical challenges such as high variability in NO measurements, small sample sizes, and complex interactions with other signaling molecules, are investigated along with approaches to alleviate these limitations. New computational techniques, including machine learning, integrative omics approaches, and network-based systems biology, present commanding outlines for identifying NO-mediated regulatory mechanisms. Furthermore, we underscore the necessity for interdisciplinary collaboration, open science practices, and standardized protocols to improve the reproducibility and dependability of NO research. By combining robust statistical methods with advanced computational tools, researchers can gain enhanced insights into NO biology and its effects on plant adaptation and resilience.

## 1 Introduction: context and significance of nitric oxide in plant biology

### 1.1 Overview of nitric oxide in plant systems

Nitric oxide (NO) is a pivotal signaling molecule that plays diverse roles in plant physiological and developmental processes, including seed germination, root development, vascular patterning, and stomatal closure (Kumar and Ohri, 2023; Pande et al., 2021). It modulates gene expression related to oxidative stress, hormone signaling, and immune responses. NO’s interaction with reactive oxygen species (ROS) and phytohormones like auxin, ethylene, and abscisic acid contributes to the fine-tuning of plant growth and stress responses (Khan et al., 2023).

Recent advances in imaging technologies have further revealed the complexity of NO signaling. A novel above ground whole-plant live imaging method demonstrated the intricate spatial and temporal relationship between NO and hydrogen peroxide, providing real-time visualization of their dynamic cross-talk in response to environmental cues (Mohanty et al., 2025).

NO also participates in cross-talk with other signaling molecules, influencing plant responses to light, temperature, and water availability. Its involvement in processes such as programmed cell death (PCD), protein synthesis, and photosynthesis highlights the need for integrated approaches—including genomics, transcriptomics, and proteomics—to understand its complex regulatory networks (Ombale et al., 2025).

### 1.2 Nitric oxide and its signaling pathways in plants

NO regulates key physiological activities such as germination, flowering, and senescence, and enhances tolerance to biotic and abiotic stressors (Gupta et al., 2022; Nabi et al., 2021). It influences stomatal activity, osmolyte accumulation, and stress-responsive genes expression. During biotic stress, NO boosts antimicrobial compound production and reinforces cell walls. It also promotes cell division, elongation, and differentiation—such as through lateral root formation with auxin signaling (Zhao et al., 2021).

### 1.3 Challenges in nitric oxide research

Despite its importance, NO research faces several challenges due to its reactive and transient nature. NO readily reacts with ROS, metals, and thiol groups to form RNS, such as peroxynitrite and S-nitrosothiols, which are key signaling intermediates but complicate detection and interpretation (Astier et al., 2018).

Moreover, NO is produced via both enzymatic (e.g., nitrate reductase, nitric oxide synthase-like enzymes) and non-enzymatic pathways, with production varying by developmental stage and environment (Allagulova et al., 2023b). These factors result in spatiotemporal fluctuations that limit measurements reproducibility. Detection techniques—such as chemiluminescence and fluorescent probes—often suffer from specificity issues or introduce artifacts, reinforcing the need for well-designed experiments and robust data interpretation.

### 1.4 Role of statistical approach in NO research

Statistical tools are crucial for managing the variability and complexity of NO-related data. Techniques for normalization, error quantification, and noise reduction enhance data reliability. Multivariate methods such as principal component analysis (PCA) and partial least squares regression (PLSR) help identify meaningful patterns in complex datasets (Gholami et al., 2024).

Mixed-effects models accommodate both fixed and random effects (e.g. genotypes, tissue type, or environment), improving parameter precision (Tahjib-Ul-Arif et al., 2022). Meta-analysis aggregate data across studies, yielding more robust conclusions and mitigating study-specific biases (Liu et al., 2023).

### 1.5 Significance of integrating statistics in NO research

This review highlights the value of integrating statistical methods into NO research and provides guidance on their application in plant biology. By leveraging tools such as multivariate analysis, mixed-effects models, and meta-analysis, researcher can improve measurement precision and reproducibility.

We begin with core statistical concepts—hypothesis testing, normalization, and error analysis—and advance to time-series analysis and cross-study synthesis. Emphasis is placed on open source-platforms like R and Python for data analysis and visualization. Best practices in data management and sharing are also discussed to promote transparency. Ultimately, this review aims to equip researchers with quantitative tools necessary to address NO-related challenges and gain deeper insight into its roles in plant development and stress adaptation.

## 2 Experimental design and data collection in NO research

NO is a transient and reactive signaling molecule involved in diverse physiological processes in both plants and photosynthetic microorganisms such as microalgae. Owing to its low abundance, short half-life, and high reactivity with cellular components, robust experimental design and accurate data collection are essential for detecting, quantifying, and interpreting NO dynamics. This section outlines key considerations in sampling strategies, detection techniques, and managing variability—each of which is critical for generating reproducible, statistically sound, and biologically meaningful NO measurements.

### 2.1 Sampling strategies for NO measurement

Accurate sampling is foundational to NO quantification. In controlled environments involving homogeneous populations—such as genetically identical *Arabidopsis thaliana*—random sampling ensures unbiased data representation (Lohr, 2021). However, in studies incorporating heterogeneity in tissue types (e.g., roots vs. leaves), developmental stages (e.g., seedling vs. flowering), or environmental gradients (e.g., light exposures), stratified sampling provides superior accuracy. Stratification involves dividing the population into relevant subgroups (strata), and drawing random samples from each, thereby reducing sampling error and enhancing biological relevance (Singh et al., 1996).

A critical component of experimental design is determining the appropriate sample size. Instead of using arbitrarily replicate numbers, researchers should conduct a prior power analysis to define the minimal number of biological replicates required to detect a biologically significant effect size at specified significant level (typically α = 0.05) and power (commonly 0.8). For example, in an experiment comparing NO accumulation between wild-type and nitrate reductase-deficient mutants under salt stress, power analysis can determine whether three or more biological replicates are sufficient to detect a 20% difference with acceptable confidence (Ryan, 2013).

### 2.2 Detection methods and their statistical implications

NO detection in plant systems necessitates methods that are both sensitive and selective, given the molecule’s short-lived nature and low concentrations. The detection techniques selected directly influence both the accuracy and the interpretability of results under physiological and stress conditions. Three principal platforms dominate current NO research: chemiluminescence, fluorescence-based probes, and electron paramagnetic resonance (EPR), each bearing distinct advantages, limitations, and statistical requirements.

Chemiluminescence relies on the reaction between NO and ozone to generate photon emission, allowing highly sensitive quantification of gaseous NO. It is particularly suitable for measuring NO emission from leaves or aqueous plant samples under stress. However, its limitation lies in its lack of spatial resolution and applicability to bulk measurements. To ensure reproducibility, frequent calibration using standard NO donors such as DEA-NONOate is necessary (Sparacino-Watkins and Lancaster, 2021).

Fluorescence probes—including DAF-FM and DAR-4M—enable real-time imaging of intracellular NO and offer both spatial and temporal resolution. These cell-permeable dyes form fluorescence adducts upon reacting with NO, facilitating *in situ* quantification. However, probe performance can be influenced by pH, temperature, and interactions with other ROS and RNS. Adequate controls—such as pH-stable analogs and NO-deficient mutants—are essential. Calibration with NO donors can assist in quantification, but potential non-specificity must be accounted for (Gupta et al., 2020).

EPR provides a highly specific detection of NO trapping it with paramagnetic agents (e.g., Fe^2+-^ diethyldithiocarbamate). It enables differentiation of NO from other radicals and quantification *in vivo*. While highly accurate, EPR is limited by its need for specialized instrumentation. Statistical reproducibility is enhanced through the use of internal standards and repeated independent replicates (Calvo-Begueria et al., 2018).

For all detection methods, statistical calibration is essential. Calibration curves using NO donors establish the linear relationship between signal output and NO concentration, with regression analysis providing coefficient of determination (R²) to assess the goodness-of-fit (Mohamed et al., 2022). In addition, defining the method’s limit of detection (LOD) and limit of quantification (LOQ) ensures data are interpreted within the valid operational range—especially critical when measuring physiological NO concentrations in the nanomolar range (Wood, 2015).

### 2.3 Managing variability and noise in NO data

NO data are frequently affected by variability from both biological and technical sources (Archer, 1993). Biological variation may arise from genotype, tissue type, developmental stage, or environmental conditions (e.g., light, nutrients), while technical variability often stems from sample handling, probe stability, or detector sensitivity. Mitigating this variability is vital for data reliability and biological interpretation.

Robust experimental controls play a central role in reducing confounding factors. Positive controls, such as NO donors (e.g., SNP), confirm detection capability; scavengers (e.g., CPTIO) validate signal specificity; and enzymatic inhibitors (e.g., tungstate for nitrate reductase) help dissect NO biosynthesis pathways (Pirooz et al., 2021; Song et al., 2025). Genetic tools, such as *nia1*/*nia2* in *A. thaliana*, serve as NO-dependent controls for validating physiological responses including root growth or stomatal conductance (Hao et al., 2010).

Replication is also critical. While a minimum of three biological replicates is often cited, optimal replication should be guided by power analysis tailored to the expected effect size and experimental noise (Gaskin and Happell, 2014). Technical replicates help assess the precession of measurement tools and identify variability arising from instrument drift or procedural inconsistencies.

Quantitative metrics further support the evaluation of data quality. The coefficient of variation (CV)—defined as the ratio of the standard deviation (SD) to the mean—is widely used to assess consistency across technical replicates. A CV below 10% generally indicates stable measurements, while values above 20% may signal the need for protocol refinement (Moczko et al., 2016). The interclass correlation coefficient is also valuable when comparing measurements across methods or runs; an ICC > 0.75 denotes good reliability, while ICC < 0.75 suggests poor reproducibility (Krishnan et al., 2015).

Collectively, these practices—statistical controls, genetically informed tools, rigorous replication, and variability metrics—form the basis for high-quality NO research. They allow researchers to distinguish true biological variation from experimental noise, thereby strengthening the validity and reproducibility of conclusions regarding NO function in plant biology.

**Table 1** summarizes the key statistical and methodological considerations across sampling, detection, and data management for NO research in plants. Each entry includes a description, example, and reference to aid in experimental planning and data interpretation.

**Table 1:** Statistical considerations in experimental design data collection for NO research in plants.

Section	Key considerations	Details/examples	References
2.1 Sampling stress for NO measurement	Random sampling	Ensures equal chance for each individual in the population to be selected	(Lohr, 2021)
	Stratified sampling	Divides population into subgroups based on characteristics (e.g., tissue type, developmental stage) for more accurate representation of NO data	(Singh and Chaudhary, 1981)
	Sample size determination	Power analysis to calculate minimum sample size required to detect significant effects with desired confidence	(Ryan, 2013)
2.2 Detection methods and their statistical implications	Chemiluminescence	Measures light emitted during the reaction of NO with ozone. Highly sensitive and specific for NO quantification	(Sparacino-Watkins and Lancaster, 2021)
	Fluorescence probes	Probes like DAF-FM and DAR-4M allow real-time NO measurements in living tissues, though can be influenced by environmental factors (e.g., pH, temperature)	(Goshi et al., 2019)
	Electron paramagnetic resonance	Used for direct measurement of NO and other free radicals, providing high sensitivity	(Calvo-Begueria et al., 2018)
	Calibration curves	Used to relate the measured signal (e.g., fluorescence intensity) to NO concentration	(Hetrick and Schoenfisch, 2009)
	Linear regression and R^2^	Linear regression analysis to generate calibration curves and assess the goodness of fit (R^2^ value) for accurate measurements	(Ebrahimzadeh et al., 2010)
	Limit of detection and limit of quantification	Establishes sensitivity and reliability of detection methods	(Hetrick and Schoenfisch, 2009)
2.3 Handling variability and noise in NO data	Control experiments	Use of NO scavengers or inhibitors to ensure specificity of NO measurements	(Astier et al., 2018)
	Replicates	Incorporating multiple measurements of the same condition to assess the consistency and reliability of data	(Arasimowicz-Jelonek et al., 2009)
	Coefficient of variance	Measures relative variability; low CV indicates high precession, while high CV suggests more variability that may need further investigation	(Canchola et al., 2017)
	Intraclass correlation	Evaluates reliability of repeated measurements or agreement between detection methods; higher ICC values indicate greater consistency and reliability	(Paciência et al., 2021)

## 3 Statistical tools for analyzing NO data

NO acts as a multifunctional signaling molecule in plant biology, influencing a broad range of physiological and stress-related processes—from drought and salinity tolerance to immune responses and development (Khan et al., 2021, 2019). However, its reactive, transient nature, and its involvement in complex biosynthetic and scavenging pathways complicate data interpretation. Robust statistical approaches are thus essential for analyzing NO-related datasets in a biologically meaningful and reproducible manner. This section outlines key statistical tools used in NO research, from basic descriptive measures to advanced modeling multivariate and time-series analysis, offering a framework to support reliable data interpretation.

### 3.1 Descriptive statistics for summarizing NO levels

Descriptive statistics are foundational for summarizing and exploring NO measurements. While the mean is commonly used to report average NO levels across replicates, the median often provides a more robust estimate of central tendency in skewed datasets—frequently encountered under stress treatments (Huang and Li, 2014; Zeiger et al., 2011). Measures of variability such as SD and CV helps assess the dispersion and reproducibility of NO levels within and between the experimental groups (Karvonen and Lehtimäki, 2020; Rizwan et al., 2018).

Visualization tools enhance interpretability. Box plots are particularly informative for comparing NO distributions across treatments, interquartile ranges, and outliers (León et al., 2016; Tang et al., 2019). For instance, León et al. (2016) used box plots to depict transient shifts in lipid metabolism and chlorophyll degradation in *A. thaliana* following NO treatment. Bar graphs, typically displaying group means and associated error bars (SD or SE), facilitate quick comparisons of NO levels across genotypes or treatments (Kaya et al., 2020).

### 3.2 Inferential statistics for hypothesis testing

Inferential statistics are central to determining whether observed differences in NO levels are statistically significant. t-test are widely employed to compare NO concentrations between two groups—e.g., wild-type vs. mutant plants under salinity stress (Hao et al., 2010; Shu et al., 2025). Independent t-tests apply to unpaired data (distinct individuals), while paired t-tests are appropriate for repeated measurements on the same sample.

For comparisons involving more than two conditions, one-way Analysis of Variance (ANOVA) assess whether NO levels differ significantly among groups (e.g., varying SNP concentrations). Two-way ANOVA, is especially valuable when testing interaction effects, such as genotype × treatment combinations influencing NO synthesis (Paul et al., 2023; Shu et al., 2025). Following ANOVA, *post-hoc* tests like Tukey’s Honestly Significant Difference (HSD) or Bonferroni correction are used to identify specific pairwise differences while minimizing Type I error risk (Agbangba et al., 2024; Ozdemir et al., 2024).

Regression analysis is commonly applied to explore associations between NO levels and predictor variables, such as light intensity or physiological responses (e.g., stomatal closure). Linear regression suits simple trends, whereas nonlinear models may better represent feedback-regulated or dose-response relationships in NO biosynthesis (Olin et al., 2004; Tahjib-Ul-Arif et al., 2022).

### 3.3 Multivariate analysis for complex data sets

Given NO’s integration into multi-layered plant signaling networks, multivariate techniques are indispensable for interpreting complex, high-dimensional datasets. PCA is widely used in NO-related metabolomic or transcriptomic studies to reduce dimensionality and highlight dominant trends. Nabati et al. (2024), for example, employed PCA to dissect stress responses in cold-exposed potato seedlings, identifying strong correlation between NO-related variables and treatments regimes.

Cluster analysis, including k-means clustering, enables classification of experimental samples based on NO accumulation and related phenotypes. Lv et al. (2022) grouped wheat genotypes by NO profiles and drought tolerance, revealing functionally distinct stress-resilient clusters.

Structural Equation Modeling (SEM) provides a powerful approach for inferring causal relationships among interconnected variables—e.g., NO levels, ROS production, and enzymatic responses (Han et al., 2021; Yu et al., 2021). Yu et al. (2021) applied SEM to examine nitrogen acquisition in *Mikania micrantha*, revealing that NO-mediated microbial interactions enhanced rhizospheric nitrogen cycling.

### 3.4 Time-series analysis for dynamic NO studies

NO production is inherently dynamic, especially in response to biotic or abiotic triggers (Khan et al., 2023). Time-series analysis allows researchers to model these fluctuations and detect patterns over time. Basic trend analysis can identify directional changes in NO levels following treatments (e.g., flg22-induced defense response). More advanced methods like Autoregressive Integrated Moving Average (ARIMA) are used to model temporal dependencies and predict future NO levels values, such as those associated with circadian rhythms or developmental transitions (Al Yammahi and Aung, 2023).

Wavelet analysis has emerged as a valuable tool for detecting transient or oscillatory NO signals—especially in spatial resolved contexts like root tips or stomata. For instance, Wang et al. (2024) applied continuous wavelet analysis (CWA) to canopy reflectance data in maize, identifying specific spectral features that accurately predicted nitrogen indices. These approaches hold promise for quantifying real-time, environmentally responsive signals.

### 3.5 Dealing with confounding factors in complex NO pathways

NO biosynthesis and function are intricately connected to other signaling pathways, involving ROS, hormones, and environmental cues (León and Costa-Broseta, 2020). Therefore, careful statistical control for confounding variables is critical. Regression models should incorporate covariates (e.g., light intensity, temperature) known to influence both NO and the outcomes of interest. Similarly, factorial designs and two-way ANOVA can help disentangle the individual and interactive effects of multiple factors, such as genotype and treatment.

**Table 2** summarizes the key statistical techniques discussed in this section, offering guidance for their application in NO-focused research. This structured approach empowers researchers to select context-appropriate analysis, ensuring scientifically sound and reproducible conclusions in the rapidly advancing field of plant NO biology.

**Table 2:** Statistical methods for analyzing NO data in plants.

Section	Statistical method	Key considerations	References
3.1 Descriptive statistics for summarizing NO levels	Mean	Represents the average NO level in a dataset. Sensitive to extreme values	(Liu et al., 2016)
	Median	Middle value in an order dataset. Useful for skewed distributions.	(Huang and Li, 2014)
	Standard deviation	Measures variability of NO levels around the mean. Higher SD indicates greater spread.	(Karvonen and Lehtimäki, 2020; Rizwan et al., 2018)
	Bar graphs	Used for comparing mean NO levels across different groups	(Kaya et al., 2020)
	Box plots	Visualize distribution, median, quartiles, and outliers in NO data	(León et al., 2016; Tang et al., 2019)
3.2 Inferential statistics for hypothesis testing	t-test (Independent/paired)	Compare NO levels between two groups; independent for unpaired data, paired for repeated data	(Hao et al., 2010; Shu et al., 2025)
	Analysis of variance	One-way ANOVA for single-factor comparisons; two-way ANOVA for interaction effects	(Paul et al., 2023; Shu et al., 2025)
	Regression analysis	Examines relationships between NO levels and predictor variables; linear and non-linear models	(Tahjib-Ul-Arif et al., 2022)
	*Post-hoc* test (Turkey’s HSD, Bonferroni correction)	Identifies specific group differences while controlling for Type I errors	(Agbangba et al., 2024; Ozdemir et al., 2024)
3.3 Multivariate analysis for complex data sets	Principal component analysis	Reduces dimensionality of NO datasets, identifies dominant patterns	(Nabati et al., 2024)
	Cluster analysis (k-means clustering)	Groups similar NO data points based on predefined criteria. Useful for phenotypic classification	(Lv et al., 2022)
	Structural equation modeling	Models causal relationships among NO levels, environmental factors, and biological responses	(Han et al., 2021; Yu et al., 2021)
3.4 Time-series analysis for dynamic NO studies	Trend analysis	Identifies long-term patterns and fluctuations in NO levels	(Al Yammahi and Aung, 2023)
	Autoregressive integrated moving average	Captures temporal dependencies; predicts future NO levels	(Al Yammahi and Aung, 2023)
	Wavelet analysis	Detects transient changes and periodicities in NO fluctuations	(Wang et al., 2024)
3.5 Dealing with confounding factors	Covariate inclusion in regression	Controls for external variables (e.g., light, temperature) influencing NO and outcomes	(León and Costa-Broseta, 2020)
	Factorial design/Two-way ANOVA	Disentangles interaction effects (e.g., genotype × treatment)	(Paul et al., 2023)

## 4 Applications of statistical methods in NO research

NO plays a multifaceted role in plant biology, acting as a signaling molecule in processes ranging from stress responses to development. Statistical methodologies have significantly advanced our understanding of NO’s roles by enabling rigorous data analysis, pattern recognition, and hypothesis testing. This section presents representative examples of how statistical tools are applied in NO research across different physiological contexts, including abiotic stress tolerance, plant-pathogen interactions, and growth and development. A summary of statistical techniques, NO detection methods, and their respective advantages, limitations, and software tools is provided in **Table 3**.

**Table 3:** Overview of NO detection methods, statistical tools, and applications.

NO detection method	Type	Strength	Limitations	Common statistical tools	Software tools
DAF-FM DA fluorescence	Semi-quantitative	High-sensitivity, cell-level resolution	Snapshot data, photobleaching	ANOVA, t-tests, correlation	ImageJ, R, SPSS
Griess assay	Quantitative	Simple, cheap	Measures NO_2_- not NO directly	Linear regression, PCA	Excel, R
Electrochemical probes	Real-time	Dynamic monitoring possible	Expensive, low spatial resolution	Time-series, wavelet, SEM	MATLAB, Python, R
Imaging (confocal)	Visual	Spatial dynamics	Semi-quantitative	PCA, cluster analysis	Fiji, Python (OpenCV)
Omics integration (e.g., RNA-seq)	Multidimensional	Systems-level insights	Complex modeling required	ML, regression, clustering	TensorFlow, Scikit-learn, R

### 4.1 NO in abiotic stress tolerance

NO plays a pivotal role in enhancing plant resilience against abiotic stresses such as drought, salinity, and heavy metal toxicity. Researchers frequently apply statistical methods such as ANOVA, correlation, and regression to quantify NO responses under stress and to elucidate its interactions with physiological parameters. In drought stress studies, significant increases in NO accumulation have been observed in stressed plants compared to well-watered controls. For instance, ANOVA revealed elevated NO levels in wheat and rice under drought, while linear regression models showed a strong positive correlation between NO content and relative water content (RWC), with one wheat study reporting a correlation coefficient of r = 0.85 (Allagulova et al., 2023a). This indicate that NO contributes to drought resilience by helping maintaining cellular hydration.

Under salinity stress, correlation analysis has revealed associations between NO levels and antioxidant enzyme activities such as superoxide dismutase (SOD), catalase (CAT), and ascorbate peroxidase (APX), indicating that NO supports cellular defense by mitigating oxidative damage (Khan et al., 2020). Similarly, in heavy metal stress conditions, regression models have linked increased NO levels with reduced cadmium accumulation and enhanced expression of stress-responsive genes, highlighting NO’s role in metal detoxification and defense (Liu et al., 2023).

To perform such analysis, researchers commonly utilize software platforms such as R and IBM SPSS statistics. R is a free, open-source environment supporting a wide array of statistical techniques and customizable data visualizations. It is particularly suited for complex modeling and reproducible workflows. Key R packages used in NO research include, ggplot2 (https://ggplot2.tidyverse.org) (for high-quality plots), lme4 (https://cran.r-project.org/web/packages/lme4/index.html) (for linear and mixed-effects models), and Hmisc (https://cran.r-project.org/web/packages/Hmisc/index.html) and corrplot (https://cran.r-project.org/web/packages/corrplot/index.html) (for descriptive statistics and correlation matrices).

For those performing a graphical interface, SPSS offers intuitive tools for ANOVA, correlation, and regression without coding, making it especially useful for researchers unfamiliar with programming.

By leveraging these tools, plant scientists can uncover insights into how NO functions under abiotic stress. Quantitative methods strengthen conclusions and enable predictive models for stress tolerance, ultimately supporting crop improvement programs.

### 4.2 NO in plant-pathogen interactions

NO is central in plant defense, particularly against microbial pathogens. It acts alongside ROS and defense-related phytohormones such as salicylic acid (SA) to mediate local and systemic resistance. Quantifying and modeling NO’s role in immunity requires statistical tools ranging from comparative tests to multivariate and causal analyses.

Methods like t-tests and ANOVA evaluate NO level differences between infected and control plants. For example, Clarke et al. (2000) reported significantly increased NO in *A. thaliana* leaves upon *Pseudomonas syringae* infection. When multiple treatments or time points are involved, *post-hoc* tests such as HSD identify specific differences. These can be efficiently conducted using JASP (https://jasp-stats.org/), an open-source platform offering interactive visualizations and a drag-and-drop interface.

In complex experiments, multivariate approaches like PCA and cluster analysis identify patterns across variables or genotypes. Tian et al. (2020) used PCA and weighted gene co-expression network analysis (WGCNA) to distinguish resistant and susceptible rice cultivars during rice blast infection, identifying gene modules linked to differential responses. The FactoMineR (https://cran.r-project.org/web/packages/FactoMineR/index.html) package in R, along with factoextra (https://cran.r-project.org/web/packages/factoextra/index.html), supports such analyses and enhance visual output.

To examine directional and causal relationships among NO, ROS, and gene expression, SEM is effective. SEM has been used to model interactions involving NO production, oxidative bursts, and defense gene activation (Yoshioka et al., 2009). R’s lavaan package (https://lavaan.ugent.be/) provides intuitive syntax and diagnostics, while semplot aids visualization. GUI-based tools like AMOS (https://www.ibm.com/products/structural-equation-modeling-sem) and JASP (https://jasp-stats.org/) also support SEM for users performing visual workflows.

These statistical strategies support a mechanistic understanding of NO in pathogen defense, enabling researchers to progress from descriptive to predictive and causal insights using accessible, interdisciplinary tools.

### 4.3 NO in plant growth and development

NO regulates processes such as seed germination, root architecture formation, and flowering transitions. In the context of seed germination, logistic regression models have been widely used to analyze the probability of seed germination across different NO concentration gradients. Recent reviews, such as Zhang et al. (2023), have highlighted that germination rates often exhibit a nonlinear response to NO, with optimal concentrations markedly enhancing germination success compared to both lower and higher levels, suggesting a tightly regulated dose-dependent effect.

Similarly, NO’s influence on root development has been quantitatively assessed using regression analyses and ANOVA. Research by Zhao et al. (2021), revealed that NO application has a statistically significant, dose-dependent effect on lateral root formation. Lower concentrations typically promote lateral root emergence, whereas excessive NO can inhibit root elongation, illustrating the need for finely balanced NO signaling during root system architecture development.

In addition to early developmental stages, NO has been implicated in the regulation of flowering. Time-series and wavelet analysis have uncovered periodic fluctuations in NO levels that coincide with floral transition stages. For example, Zhang et al. (2019) demonstrated that NO and nitrogen metabolites regulate flowering in *A. thaliana* through distinct pathways, with NO acting to delay floral transition via modulation of FLOWERING LOCUS T (*FT*) expression—highlighting its function as a temporal regulator in flowering control.

To analyze such dynamic developmental data, time-series approaches have become increasingly valuable. Software tools such as MATLAB (https://www.mathworks.com/products/matlab.html), R packages like forecast (https://cran.r-project.org/web/packages/forecast/index.html) and TSA (Time Series Analysis; https://cran.r-project.org/web/packages/TSA/index.html), and Python libraries such as statsmodels (https://www.statsmodels.org/stable/index.html) and PyWavelets (https://pywavelets.readthedocs.io/en/latest/) offer robust frameworks for modeling time-dependent biological phenomena. These tools allow researchers to detect underlying trends, periodicities, and complex temporal interactions, providing deeper insight into the dynamic regulatory roles of NO throughout plant development.

### 4.4 Challenges and emerging approaches

Despite considerable advances, NO research in plant biology continues to face significant methodological challenges. A primary issue concerns the quantification of NO levels within biological tissues. Currently, most experimental studies rely on fluorescent probes such as DAF-FM DA, which, although widely used, provide only semi-quantitative and point-in-time measurements. These methods offer limited temporal resolution and are prone to artifacts, making it difficult to accurately capture the transient and dynamic nature of NO signaling (Gross and Durner, 2016). Given NO’s rapid turnover and reactive behavior, real-time, *in vivo* monitoring remains a major technical hurdle in the field.

These quantification limitations have important statistical implications. Many datasets generated from NO experiments represent static, single-time-point snapshots rather than continuous or longitudinal data. This restricts the ability to model temporal dynamics of NO, such as peak-through fluctuations similar to those observed in ROS signaling. Without accounting for time-dependent changes, important aspects of NO’s regulatory roles may be overlooked or misrepresented, leading to oversimplified interpretations.

To address these challenges, emerging experimental approaches are being recommended. Where feasible, experimental design should incorporate repeated measurements over time to better capture the dynamic profile of NO signaling. This would allow researchers to treat time as an explicit factor in their analysis. Statistical models such as mixed-effects models, nonlinear regression, or generalized additive models are particularly suited for handling complex, time-series data with repeated measures. These approaches can account for both fixed effects (e.g., treatment conditions) and random effects (e.g., variation between biological replicates) while modeling nonlinear, dynamic trends. In parallel, advancements in imaging technologies and biosensors may eventually allow more accurate real-time tracking of NO in living tissues, opening new possibilities for dynamic statistical modeling (Saini et al., 2023). Integrating these methodological improvements will enhance the biological interpretation of NO data and strengthen the statistical rigor of future studies.

### 4.5 The role of artificial intelligence and interdisciplinary collaboration

AI technologies, particularly machine learning (ML) and deep learning (DL) approaches, are increasingly recognized as transformative tools in NO research. ML algorithms such as random forests, support vector machines (SVMs), and artificial neural networks have been successfully applied to predict plant stress outcomes or classify physiological responses based on NO profiles and related biochemical markers. For example, an ML model trained on integrated multi-omics data—including NO levels, transcriptomic profiles, and ROS signals—has demonstrated high accuracy in classifying plant stress status, enabling earlier and more precise detection of stress responses (Hesami et al., 2022). Accessible platforms such as Scikit-learn (https://scikit-learn.org/stable/), TensorFlow (https://www.tensorflow.org/), and WEKA (https://www.cs.waikato.ac.nz/ml/weka/) provide researchers with user-friendly environments to build, train, and validate these predictive models, even with complex and high-dimensional datasets (Mirani et al., 2021).

The successful implementation of AI in NO research increasingly depends on effective interdisciplinary collaboration. Biologists contribute essential expertise in experimental design, physiological interpretation, and domain-specific knowledge, ensuring that datasets are biologically meaningful and that hypotheses are grounded in mechanistic understanding. Statisticians play a critical role in developing rigorous analytical frameworks, validating models, and ensuring appropriate hypothesis testing, thus minimizing biases and overfitting risks. Meanwhile, data scientists bring specialized skills to construct robust, scalable data pipelines and optimize ML models for high-dimensional, heterogeneous biological datasets (John, 2024). Such integrated, cross-disciplinary efforts are not only enhancing the accuracy and interpretability of AI-driven NO research but also accelerating discoveries in plant biology under complex environmental conditions. For further detail, please see **Table 3**.

## 5 Challenges and limitations in statistical analysis of NO data

While statistical tools are indispensable for analyzing NO data, researchers often encounter challenges that can compromise the accuracy and reliability of their findings. These challenges include high variability in NO measurements, small sample sizes, and complex interactions among biological variables. Addressing these issues through careful experimental design and appropriate statistical strategies is essential for robust and meaningful NO research.

### 5.1 High variability and noise in NO measurements

Quantifying NO in plants remains challenging due to high biological variability and technical noise introduced by experimental conditions, detection methods, and biological heterogeneity. Environmental factors such as temperature, humidity, and light intensity can significantly influence endogenous NO production, while technical differences between detection methods—including fluorescence probes and chemiluminescence assays—often yield inconsistent results. Biological variability across tissues, developmental stages, and genotypes further compounds the challenges.

To enhance reproducibility, researchers should adopt standardized protocols for NO measurement. Resources such as the open-access Guidelines for the Measurement of NO in Biological Samples (Wink et al., 2011) provide detailed recommendations on probe calibration, autofluorescence controls, and validation steps. Strategies such as using NO scavengers (e.g., cPTIO) to confirm probe specificity, combining complementary methods (fluorescence and chemiluminescence), and strictly regulating growth conditions (temperature, humidity, light) are highly recommended.

Increasing biological replicates, carefully planning sampling times, and applying data-smoothing techniques such as moving averages or LOESS smoothing can further reduce experimental noise. For instance (Jacobson et al., 2018), demonstrated that using DAF-FM DA under rigorously controlled conditions minimized variability and enabled reliable detection of NO dynamics during drought stress responses. Standardization and methodological rigor are thus critical for extracting meaningful biological insights from NO data.

### 5.2 Small sample sizes and their impact on statistical power

Studies on NO function, particularly in context such as seed germination and early development, often face limitations due to small sample sizes. Insufficient sample sizes severely compromise statistical power, increasing the likelihood of Type II errors (failing to detect true biological effects). Moreover, small datasets are particularly sensitive to outliers and random variability, which can mislead interpretations.

A strong example comes from Liu et al. (2019), where researchers examined exogenous NO effects on seed germination using three biological replicates of 100 seeds per treatment group—totaling 8,400 seeds across all treatments. This substantial sample size allowed for robust statistical analyses, clearly revealing dose-dependent effects of NO.

To avoid pitfalls associated with small sample sizes, researchers should conduct power analyses before experimentation. Free tools such as G*Power Faul et al. (2007) can help estimate the minimum number of replicates needed to detect biologically meaningful differences with a desired confidence level. When increasing sample size is impractical, meta-analyses pooling data from independent studies can strengthen statistical power and improve generalizability. Prioritizing sample size and statistical rigor is vital for producing reliable, reproducible conclusions in NO research.

### 5.3 Complex interactions and confounding factors

NO functions within a complex signaling network involving ROS, reduced glutathione (GSH), hydrogen sulfide (H_2_S), phytohormones, and post-translational modifications such as S-nitrosylation and tyrosine nitration. Environmental variables (e.g., microbial activity, soil composition) and genetic factors further modulate these interactions, making it difficult to isolate NO-specific effects.

A major analytical concern in such systems is multicollinearity, where strong correlations among predictor variables (e.g., NO and ROS levels) inflate variance and obscure true biological relationships. To address these complexities, researchers should employ multivariate statistical techniques. PCA and SEM are particularly effective for disentangling independent variables and identifying causal relationships. Open-source tools like FactoMineR (Lê et al., 2008) and lavaan in R (Rosseel, 2012) provide accessible frameworks for these analyses.

Careful experimental design can also minimize confounding effects. Key strategies include randomization, blocking by growth stage or genotype, and factorial designs to assess interactive effects between NO and other factors. Additionally, sensitivity analyses, where models are tested under varying assumptions, can help validate the robustness of statistical conclusions.

Concrete examples illustrate the importance of these approaches. In a meta-analysis, Tahjib-Ul-Arif et al. (2022) systematically examined the role of exogenous NO in salinity stress tolerance, carefully separating NO-specific effects from those mediated by interacting antioxidants like GSH and hormonal pathways. Their work highlights the need for robust multivariate statistical frameworks to parse complex signaling networks.

Recognizing the dynamic and interconnected nature of NO signaling—and employing rigorous experimental and analytical approaches—is essential for accurately uncovering NO’s diverse roles in plant biology.

## 6 Emerging statistical techniques in NO research

NO plays a pivotal role in plant biology, acting as a signaling molecule in diverse physiological processes including growth, development, immunity, and stress responses. However, its reactive nature low concentrations, and spatiotemporal variability pose substantial challenges for accurate detection and mechanistic interpretation. Advances in statistical methodologies and computational biology now offer powerful tools to address these complexities, enabling more robust modeling, integration, and interpretation of NO-mediated processes. This section critically examines emerging approaches, focusing on ML, integrative omics, and systems biology frameworks, while underscoring the need for methodological rigor and standardized protocols.

### 6.1 Machine learning and predictive modeling

ML approaches are increasingly utilized in plant NO research due to their capacity to analyze high-dimensional, non-linear datasets. Supervised learning algorithms such as SVMs, random forests (RFs), and artificial neural networks (ANNs) have been applied to predict physiological outcomes based on NO levels, integrating data from transcriptomic, environmental, and metabolic variables (Islam et al., 2024). These methods are particularly suited for classification tasks, such as distinguishing stress-resistant from susceptible phenotypes in crops exposed to NO donors or environmental stressors.

Understanding methods like PCA, k-means clustering, and hierarchical clustering are employed to detect hidden patterns and co-regulated gene/metabolite clusters in response to NO signaling (Pires et al., 2008). Moreover, recently DL architectures—especially convolutional neural networks (CNNs) and recurrent neural networks (RNNs)—have demonstrated potential in modeling dynamic responses to NO fluctuations over time (Gao et al., 2020), although their application remains limited due to data scarcity and lack of model interpretability.

Despite their promise, ML models require careful feature selection, model validation, and transparency. Few studies in NO biology have benchmarked ML algorithms against conventional statistical techniques or performed external validation on independent datasets. Moreover, standardization in data preprocessing and metadata documentation is essential for reproducibility and cross-study comparisons.

### 6.2 Integration of omics data

NO research increasingly leverages high-throughput omics platforms—transcriptomics, proteomics, metabolomics, and epigenomics—to elucidate molecular mechanisms of NO signaling. Integrative statistical frameworks allow researchers to unify these datasets, revealing cross-layer regulatory relationships. Weighted gene co-expression network analysis (WGCNA), partial least squares regression (PLSR), Bayesian networks, and canonical correlation analysis (CCA) have proven effective in identifying NO-responsive modules and candidate biomarkers (Jiang et al., 2023; Singh et al., 2022).

For example, transcriptomic analyses have identified NO-induced genes involved in redox regulation and hormone signaling (Hussain et al., 2016), while proteomics has uncovered NO-dependent post-translational modifications such as S-nitrosylation and tyrosine nitration (Jindal and Seth, 2023). Metabolomic profiling further links NO to alterations in primary and secondary metabolism under abiotic stress (Zhou et al., 2021). Integrative tools such as mixOmics, DIABLO, and iOmicsPASS streamline the fusion of these datasets, though biological interpretation remains limited by incomplete annotation of NO targets and contextual variability across species and stress conditions.

Emerging methods like multi-omics factor analysis and regularized generalized CCA offer improved dimensionality reduction and latent variable discovery, especially for studies constrained by small sample sizes (Jiang et al., 2023). However, challenges persist in data harmonization, batch effect correction, and the need for curated NO-specific databases.

### 6.3 Network analysis and systems biology approaches

To model the complexity of the NO signaling networks, systems biology approaches—particularly network-based analysis—have emerged traction. Gene regulatory networks (GRNs), protein-protein interaction (PPI) networks, and metabolic networks collectively offer insights into NO-mediated cross-talk with other signaling molecules such as ROS, phytohormones (e.g., ABA, SA, ethylene), and calcium ions (Pavlopoulos et al., 2011; Vazquez, 2011).

Computational platforms like Cytoscape and STRING facilitate the construction and visualization of interaction networks, aiding in the identification of hub genes, network motifs, and critical nodes within NO-associated pathways. These analyses have been instrumental in delineating NO’s role in PCD, systemic acquired resistance, and stress memory (Do Amaral et al., 2020; Luo et al., 2025; Wang et al., 2023). Boolean modeling and ordinary differential equation (ODE)-based simulations provide dynamic insights into NO kinetics, its dose-dependent effects on downstream pathways, and cross-talk with other signaling molecules—offering a systems—level perspective on NO-mediated regulation (Karanam and Rappel, 2022). Recent efforts have also begun to incorporate spatial modeling approaches to better capture NO diffusion and compartmentalization within plant tissues, though these remain underexplored (Airaki et al., 2015).

While network analysis offers powerful visualization and hypothesis generation tools, most networks are inferred from static data and lack temporal resolution. Moreover, functional validation of inferred nodes and edges is often lacking, underscoring the need for experimental integration.

### 6.4 Need for experimental standardization and interdisciplinary integration

Despite advancing in statistical modeling, the accuracy of NO studies remains heavily dependent on experimental design. NO detection techniques—including electrochemical sensors, fluorescent dyes (e.g., DAF-FM DA), and chemiluminescence—vary in sensitivity, specificity, and spatiotemporal resolution. The lack of standard protocols for sample handling, NO quantification, and metadata reporting hampers data comparability and integrative analysis (Bryan and Grisham, 2007). A critical need exists for benchmarking studies comparing NO detection methods under controlled conditions.

Furthermore, interdisciplinary collaboration between plant biologists, statisticians, chemists, and data scientists is essential for advancing this field. Integrating expertise from these domains can refine experimental protocols, improve model interpretability, and ensure biologically meaningful insights from complex data.

**Figure 1:**
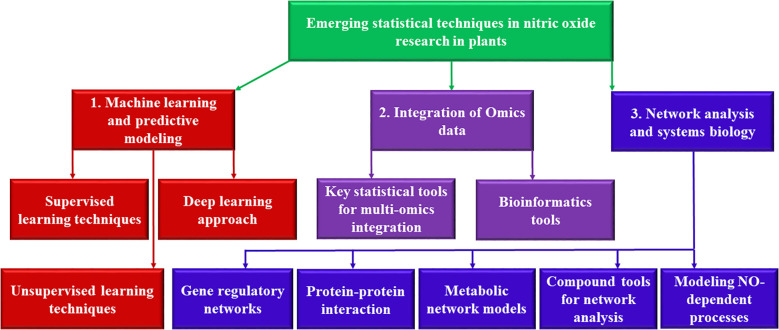
Overview of emerging statistical techniques in NO research. The figure illustrates the integration of machine learning models, multi-omics data analysis, and network analysis and systems biology approaches to uncover NO-mediated regulatory mechanisms in plants. (1) Machine learning techniques, including supervised learning (SVM, RF, ANN) and deep learning (CNN, RNN), predict plant responses to varying NO levels based on gene expression, environmental factors, and metabolic markers. (2) Integrative omics frameworks combine transcriptomics, proteomics, and metabolomics using statistical models such as WGCNA, Bayesian networks, and MOFA to identify key NO-regulated pathways. (3) Network-based systems biology approaches, including gene regulatory networks, protein-protein interaction networks, and metabolic pathway analysis, elucidate cross-talk between NO and other signaling molecules like ROS and phytohormones. Computational tools such as Cytoscape and STRING aid in network construction and visualization. These advanced methodologies enhance our understanding of NO signaling, enabling precise modeling of plant adaptation and stress responses.

**Figure 1** provides a schematic representation of the emerging statistical techniques in NO research, illustrating the interplay between ML models, integrative omics analysis, and network-based systems biology approaches. The diagram showcase how supervised learning models (SVM, RF, and ANN) and DL techniques (CNN, RNN) leverage large datasets to predict plant responses to NO levels. Additionally, it highlights the integration of multi-omics datasets using statistical tools such as WGCNA, Bayesian networks, and MOFA, which enable the identification of NO-regulated pathways. The network analysis section of the figure demonstrates how GRNs, PPI, and metabolic pathways contribute to understanding NO-mediated signaling and cross-talk with other molecules like ROS and phytohormones. Computational tools such as Cytoscape and STRING are depicted as essential for visualizing these interactions, ultimately providing deeper insights into NO-regulated biological processes.

Overall, the integration of advanced statistical techniques, ML models, and systems biology approaches is transforming NO research, enabling more accurate predictions and mechanistic insights into NO-mediated biological processes. Future advancements in computational biology are expected to further refine our ability to model NO dynamics, ultimately contributing to improved plant resilience and agricultural productivity.

## 7 Future directions and recommendations

The field of NO research has made significant strides in understanding its multifaceted roles in biological systems, ranging from cellular signaling to physiological regulation. However, several challenges remain, including the complexity of NO signaling networks, the variability in experimental methodologies, and the need for robust statistical frameworks to interpret data. To address these challenges and advance the field, future efforts should focus on interdisciplinary collaboration, open science practices, and the development of standardized protocols. Below, we outline these future directions and provide recommendations for their implementation.

### 7.1 Interdisciplinary collaboration between biologists and statisticians

The complexity of NO signaling pathways and their interactions with other molecular networks necessitates a collaborative approach that bridges biology and statistics. Biologists often generate large datasets from experiments, but the interpretation of these datasets requires advanced statistical tools to account for variability, noise, and confounding factors. Conversely, statisticians may lack the biological context needed to design appropriate models for NO research. Therefore, cross-disciplinary training and collaboration are essential to harness the full potential of NO research.

Interdisciplinary collaboration can lead to the development of novel analytical tools tailored to the unique challenges of NO research. For instance, ML algorithms and network analysis techniques can be employed to map NO signaling pathways and predict their interactions with other molecules (Guo et al., 2023). Training programs that integrate biological and statistical expertise will empower researchers to design more robust experiments and interpret data with greater accuracy. Such initiatives have already shown promise in related fields, such as genomics and systems biology (Pinu et al., 2019). By fostering collaboration, the NO research community can accelerate discoveries and improve the reproducibility of findings.

### 7.2 Open science and data sharing

The reproducibility crisis in science has underscored the need for transparency and open access to data and methodologies. In NO research, variability in experimental conditions and data analysis techniques can lead to inconsistent results. Open science practices, including the sharing of raw data, code, and protocols, can mitigate these issues and promote reproducibility.

Open data sharing allows researchers to validate findings, conduct meta-analyses, and build upon existing work. For example, publicly available datasets on NO-mediated signaling in cardiovascular systems have enabled researchers to identify novel therapeutic targets (He et al., 2022). Similarly, sharing analytical code ensures that statistical methods are transparent and reproducible. Platforms such as GitHub (https://github.com) and Zenodo (https://zenodo.org) provide accessible repositories for sharing code and data, fostering a culture of openness in the scientific community. Journals and funding agencies should incentivize open science practices by mandating data and code availability as a condition for publication and grant awards.

### 7.3 Development of standardized protocols for NO research

The lack of standardized protocols in NO research has led to inconsistencies in experimental design, data collection, and analysis. Establishing guidelines for these aspects is critical to ensure the reliability and comparability of results across studies.

### 7.4 Establishing guidelines for experimental design, data collection, and statistical analysis

Standardized protocols should address key aspects of NO research, including the quantification of NO levels, the use of appropriate controls, and the selection of statistical methods. For instance, the use of fluorescent probes for NO detection should be accompanied by calibration standards to ensure accuracy (Zhang et al., 2014). Additionally, guidelines for statistical analysis should emphasize the importance of controlling for multiple comparisons and reporting effect sizes to avoid misleading conclusions (Goshi et al., 2019; Parisi et al., 2024).

### 7.5 Mapping NO signaling networks and their interactions with other molecules

A comprehensive understanding of NO signaling requires the integration of data from diverse experimental approaches, such as proteomics, metabolomics, and transcriptomics. Standardized protocols will facilitate the integration of these datasets, enabling the construction of detailed NO signaling networks. For example, recent advances in network modeling have revealed the interplay between NO and ROS in cellular stress responses (Molassiotis and Fotopoulos, 2011). By adopting standardized protocols, researchers can systematically map these interactions and identify key regulatory nodes involved in plant development and stress responses.

## 8 Conclusions

The integration of robust statistical methods is essential for advancing our understanding of NO signaling and its multifaceted roles in plant biology. The challenges associated with NO research, including measurement variability, small sample sizes, and complex biological interactions, necessitate the use of standardized experimental protocols and advanced statistical tools. Emerging approaches such as ML, multi-omics integration, and systems biology offer promising avenues for unraveling NO-mediated regulatory networks. However, the field must also prioritize interdisciplinary collaboration between biologists and statisticians to develop tailored analytical frameworks that address the specific challenges of NO research. Additionally, fostering open science practices, including data and code sharing, will enhance reproducibility and facilitate meta-analyses across studies. As computational techniques continue to evolve, the application of innovative statistical strategies will drive new discoveries in NO biology, ultimately contributing to improved plant resilience and agricultural productivity. Future research should focus on refining predictive models, integrating diverse datasets, and developing standardized guidelines to ensure consistency and comparability in NO-related studies.

## References

[B1] Agbangba C. E. Aide E. S. Honfo H. Kakai R. G. (2024). On the use of *post-hoc* tests in environmental and biological sciences: A critical review. Heliyon 10, e25131. doi: 10.1016/j.heliyon.2024.e25131 39668858 PMC11637079

[B2] Airaki M. Leterrier M. Valderrama R. Chaki M. Begara-Morales J. C. Barroso J. B.. (2015). Spatial and temporal regulation of the metabolism of reactive oxygen and nitrogen species during the early development of pepper (Capsicum annuum) seedlings. Ann. Bot. 116, 679–693. doi: 10.1093/aob/mcv023 25808658 PMC4577988

[B3] Allagulova C. Avalbaev A. Lubyanova A. Plotnikov A. Yuldashev R. Lastochkina O. (2023a). Nitric oxide (NO) improves wheat growth under dehydration conditions by regulating phytohormone levels and induction of the expression of the TADHN dehydrin gene. Plants 12, 4051. doi: 10.3390/plants12234051 38068687 PMC10708132

[B4] Allagulova C. R. Lubyanova A. R. Avalbaev A. M. (2023b). Multiple ways of nitric oxide production in plants and its functional activity under abiotic stress conditions. Int. J. Mol. Sci. 24, 11637. doi: 10.3390/ijms241411637 37511393 PMC10380521

[B5] Al Yammahi A. Aung Z. (2023). Forecasting the concentration of NO2 using statistical and machine learning methods: A case study in the UAE. Heliyon 9. doi: 10.1016/j.heliyon.2022.e12584 PMC9922785 36793966

[B6] Arasimowicz-Jelonek M. Floryszak-Wieczorek J. Kubiś J. (2009). Involvement of nitric oxide in water stress-induced responses of cucumber roots. Plant Sci. 177, 682–690. doi: 10.1016/j.plantsci.2009.09.007

[B7] Archer S. (1993). Measurement of nitric oxide in biological models. FASEB J. 7, 349–360. doi: 10.1096/fasebj.7.2.8440411 8440411

[B8] Astier J. Gross I. Durner J. (2018). Nitric oxide production in plants: an update. J. Exp. Bot. 69, 3401–3411. doi: 10.1093/jxb/erx420 29240949

[B9] Bryan N. S. Grisham M. B. (2007). Methods to detect nitric oxide and its metabolites in biological samples. Free Radical Biol. Med. 43, 645–657. doi: 10.1016/j.freeradbiomed.2007.04.026 17664129 PMC2041919

[B10] Calvo-Begueria L. Rubio M. C. Martínez J. I. Pérez-Rontomé C. Delgado M. J. Bedmar E. J.. (2018). Redefining nitric oxide production in legume nodules through complementary insights from electron paramagnetic resonance spectroscopy and specific fluorescent probes. J. Exp. Bot. 69, 3703–3714. doi: 10.1093/jxb/ery159 29701804 PMC6022593

[B11] Canchola J. Tang S. Hemyari P. Paxinos E. Marins E. (2017). Correct use of percent coefficient of variation (% CV) formula for log-transformed data. MOJ Proteom. Bioinform. 6. doi: 10.15406/mojpb.2017.06.00200

[B12] Clarke A. Desikan R. Hurst R. D. Hancock J. T. Neill S. J. (2000). NO way back: nitric oxide and programmed cell death in Arabidopsis thaliana suspension cultures. Plant J. 24, 667–677. doi: 10.1046/j.1365-313x.2000.00911.x 11123805

[B13] Do Amaral M. N. Arge L. W. P. Auler P. A. Rossatto T. Milech C. Magalhães A. M.. (2020). Long-term transcriptional memory in rice plants submitted to salt shock. Planta 251, 1–16. doi: 10.1007/s00425-020-03397-z 32474838

[B14] Ebrahimzadeh M. Nabavi S. Nabavi S. Pourmorad F. (2010). Nitric oxide radical scavenging potential of some Elburz medicinal plants. Afr. J. Biotechnol. 9, 5212–5217.

[B15] Faul F. Erdfelder E. Lang A.-G. Buchner A. (2007). G* Power 3: A flexible statistical power analysis program for the social, behavioral, and biomedical sciences. Behav. Res. Methods 39, 175–191. doi: 10.3758/BF03193146 17695343

[B16] Gao Z. Luo Z. Zhang W. Lv Z. Xu Y. (2020). Deep learning application in plant stress imaging: a review. AgriEngineering 2, 29. doi: 10.3390/agriengineering2030029

[B17] Gaskin C. J. Happell B. (2014). Power, effects, confidence, and significance: An investigation of statistical practices in nursing research. Int. J. Nurs. Stud. 51, 795–806. doi: 10.1016/j.ijnurstu.2013.09.014 24207028

[B18] Gholami F. Hajiheidari A. Barkhidarian B. Soveid N. Yekaninejad M. S. Karimi Z.. (2024). A comparison of principal component analysis, reduced-rank regression, and partial least–squares in the identification of dietary patterns associated with cardiometabolic risk factors in Iranian overweight and obese women. BMC Med. Res. Method. 24, 215. doi: 10.1186/s12874-024-02298-z PMC11428567 39333898

[B19] Goshi E. Zhou G. He Q. (2019). Nitric oxide detection methods *in vitro* and in *vivo* . Med. gas Res. 9, 192–207. doi: 10.4103/2045-9912.273957 31898604 PMC7802420

[B20] Gross I. Durner J. (2016). In search of enzymes with a role in 3′, 5′-cyclic guanosine monophosphate metabolism in plants. Front. Plant Sci. 7, 576. doi: 10.3389/fpls.2016.00576 27200049 PMC4858519

[B21] Guo F. Yang X. Hu C. Li W. Han W. (2023). Network pharmacology combined with machine learning to reveal the action mechanism of licochalcone intervention in liver cancer. Int. J. Mol. Sci. 24, 15935. doi: 10.3390/ijms242115935 37958916 PMC10649909

[B22] Gupta K. J. Hancock J. T. Petrivalsky M. Kolbert Z. Lindermayr C. Durner J.. (2020). Recommendations on terminology and experimental best practice associated with plant nitric oxide research. New Phytol. 225, 1828–1834. doi: 10.1111/nph.16157 31479520

[B23] Gupta K. J. Kaladhar V. C. Fitzpatrick T. B. Fernie A. R. Møller I. M. Loake G. J. (2022). Nitric oxide regulation of plant metabolism. Mol. Plant 15, 228–242. doi: 10.1016/j.molp.2021.12.012 34971792

[B24] Han Z. Wang J. Xu P. Sun Z. Ji C. Li S.. (2021). Greater nitrous and nitric oxide emissions from the soil between rows than under the canopy in subtropical tea plantations. Geoderma 398, 115105. doi: 10.1016/j.geoderma.2021.115105

[B25] Hao F. Zhao S. Dong H. Zhang H. Sun L. Miao C. (2010). Nia1 and Nia2 are involved in exogenous salicylic acid-induced nitric oxide generation and stomatal closure in Arabidopsis. J. Integr. Plant Biol. 52, 298–307. doi: 10.1111/j.1744-7909.2010.00920.x 20377690

[B26] He M. Wang D. Xu Y. Jiang F. Zheng J. Feng Y.. (2022). Nitric oxide-releasing platforms for treating cardiovascular disease. Pharmaceutics 14, 1345. doi: 10.3390/pharmaceutics14071345 35890241 PMC9317153

[B27] Hesami M. Alizadeh M. Jones A. M. P. Torkamaneh D. (2022). Machine learning: its challenges and opportunities in plant system biology. Appl. Microbiol. Biotechnol. 106, 3507–3530. doi: 10.1007/s00253-022-11963-6 35575915

[B28] Hetrick E. M. Schoenfisch M. H. (2009). Analytical chemistry of nitric oxide. Annu. Rev. analytical Chem. 2, 409–433. doi: 10.1146/annurev-anchem-060908-155146 PMC3563389 20636069

[B29] Huang Y. Li D. (2014). Soil nitric oxide emissions from terrestrial ecosystems in China: a synthesis of modeling and measurements. Sci. Rep. 4, 7406. doi: 10.1038/srep07406 25490942 PMC4261933

[B30] Hussain A. Mun B.-G. Imran Q. M. Lee S.-U. Adamu T. A. Shahid M.. (2016). Nitric oxide mediated transcriptome profiling reveals activation of multiple regulatory pathways in Arabidopsis thaliana. Front. Plant Sci. 7, 975. doi: 10.3389/fpls.2016.00975 27446194 PMC4926318

[B31] Islam S. Reza M. N. Samsuzzaman S. A. Cho Y. J. Noh D. H. Chung S.-O.. (2024). Machine vision and artificial intelligence for plant growth stress detection and monitoring: A review. Precis. Agric. 6, 34. doi: 10.12972/pastj.20240003

[B32] Jacobson A. Doxey S. Potter M. Adams J. Britt D. McManus P.. (2018). Interactions between a plant probiotic and nanoparticles on plant responses related to drought tolerance. Ind. Biotechnol. 14, 148–156. doi: 10.1089/ind.2017.0033

[B33] Jiang M.-Z. Aguet F. Ardlie K. Chen J. Cornell E. Cruz D.. (2023). Canonical correlation analysis for multi-omics: Application to cross-cohort analysis. PloS Genet. 19, e1010517. doi: 10.1371/journal.pgen.1010517 37216410 PMC10237647

[B34] Jindal A. Seth C. S. (2023). “Nitric oxide mediated post-translational modifications and its significance in plants under abiotic stress,” in Nitric oxide in developing plant stress resilience (San Diego, CA, USA: Elsevier Science & Technology), 233–250.

[B35] John A. (2024). The crucial role of interdisciplinary collaboration between data scientists and biologists in developing effective predictive models (Rochester, NY, USA: SSRN).

[B36] Karanam A. Rappel W.-J. (2022). Boolean modelling in plant biology. Quantitative Plant Biol. 3, e29. doi: 10.1017/qpb.2022.26 PMC10095905 37077966

[B37] Karvonen T. Lehtimäki L. (2020). Repeatability and variation of the flow independent nitric oxide parameters. J. Breath Res. 14, 026002. doi: 10.1088/1752-7163/ab4784 31550699

[B38] Kaya C. Ashraf M. AlYemeni M. N. Corpas F. J. Ahmad P. (2020). Salicylic acid-induced nitric oxide enhances arsenic toxicity tolerance in maize plants by upregulating the ascorbate-glutathione cycle and glyoxalase system. J. hazardous materials 399, 123020. doi: 10.1016/j.jhazmat.2020.123020 32526442

[B39] Khan M. Al Azawi T. N. I. Pande A. Mun B.-G. Lee D.-S. Hussain A.. (2021). The role of nitric oxide-induced ATILL6 in growth and disease resistance in Arabidopsis thaliana. Front. Plant Sci. 12, 685156. doi: 10.3389/fpls.2021.685156 34276735 PMC8285060

[B40] Khan M. Ali S. Al Azzawi T. N. I. Yun B.-W. (2023). Nitric oxide acts as a key signaling molecule in plant development under stressful conditions. Int. J. Mol. Sci. 24, 4782. doi: 10.3390/ijms24054782 36902213 PMC10002851

[B41] Khan M. N. AlSolami M. A. Basahi R. A. Siddiqui M. H. Al-Huqail A. A. Abbas Z. K.. (2020). Nitric oxide is involved in nano-titanium dioxide-induced activation of antioxidant defense system and accumulation of osmolytes under water-deficit stress in Vicia faba L. Ecotoxicology Environ. Saf. 190, 110152. doi: 10.1016/j.ecoenv.2019.110152 31927357

[B42] Khan M. Imran Q. M. Shahid M. Mun B.-G. Lee S.-U. Khan M. A.. (2019). Nitric oxide-induced AtAO3 differentially regulates plant defense and drought tolerance in Arabidopsis thaliana. BMC Plant Biol. 19, 1–19. doi: 10.1186/s12870-019-2210-3 31888479 PMC6937950

[B43] Krishnan K. Mukhtar S. F. Lingard J. Houlton A. Walker E. Jones T.. (2015). Performance characteristics of methods for quantifying spontaneous intracerebral haemorrhage: data from the Efficacy of Nitric Oxide in Stroke (ENOS) trial. J. Neurology Neurosurg. Psychiatry 86, 1258–1266. doi: 10.1136/jnnp-2014-309845 PMC4680163 25575847

[B44] Kumar D. Ohri P. (2023). Say “NO” to plant stresses: Unravelling the role of nitric oxide under abiotic and biotic stress. Nitric. Oxide 130, 36–57. doi: 10.1016/j.niox.2022.11.004 36460229

[B45] Lê S. Josse J. Husson F. (2008). FactoMineR: an R package for multivariate analysis. J. Stat. software 25, 1–18. doi: 10.18637/jss.v025.i01

[B46] León J. Costa Á. Castillo M.-C. (2016). Nitric oxide triggers a transient metabolic reprogramming in Arabidopsis. Sci. Rep. 6, 37945. doi: 10.1038/srep37945 27885260 PMC5122866

[B47] León J. Costa-Broseta Á. (2020). Present knowledge and controversies, deficiencies, and misconceptions on nitric oxide synthesis, sensing, and signaling in plants. Plant Cell Environ. 43, 1–15. doi: 10.1111/pce.13617 31323702

[B48] Liu X. Gong D. Ke Q. Yin L. Wang S. Gao T. (2023). Meta-analysis of the effect of nitric oxide application on heavy metal stress tolerance in plants. Plants 12, 1494. doi: 10.3390/plants12071494 37050120 PMC10096531

[B49] Liu J. Xue T. Shen Y. (2019). Effect of nitric oxide on seed germination and dormancy in empress trees. HortTechnology 29, 271–275. doi: 10.21273/HORTTECH04250-18

[B50] Liu S. Yang R. Pan Y. Ren B. Chen Q. Li X.. (2016). Beneficial behavior of nitric oxide in copper-treated medicinal plants. J. hazardous materials 314, 140–154. doi: 10.1016/j.jhazmat.2016.04.042 27131454

[B51] Lohr S. L. (2021). Sampling: design and analysis (Boca Raton, FL, USA: Chapman and Hall/CRC).

[B52] Luo A. Shi C. Luo P. Zhao Z. Sun M. X. (2025). The regulatory network and critical factors promoting programmed cell death during embryogenesis. J. Integr. Plant Biol. 67, 55–70. doi: 10.1111/jipb.13795 39513658

[B53] Lv L. Chen X. Li H. Huang J. Liu Y. Zhao A. (2022). Different adaptive patterns of wheat with different drought tolerance under drought stresses and rehydration revealed by integrated metabolomic and transcriptomic analysis. Front. Plant Sci. 13, 1008624. doi: 10.3389/fpls.2022.1008624 36311061 PMC9608176

[B54] Mirani A. Memon M. S. Chohan R. Wagan A. A. Qabulio M. (2021). Machine learning in agriculture: A review. LUME 10, 229–234.

[B55] Moczko E. Mirkes E. M. Cáceres C. Gorban A. N. Piletsky S. (2016). Fluorescence-based assay as a new screening tool for toxic chemicals. Sci. Rep. 6, 33922. doi: 10.1038/srep33922 27653274 PMC5031998

[B56] Mohamed G. O. Saleh M. E. Shalaby E. A. Elsafty A. S. (2022). Using biochar to control nitric oxide air pollution. J. Physics: Conf. Ser. 23051, 012029. doi: 10.1088/1742-6596/2305/1/012029

[B57] Mohanty D. Peláez-Vico M.Á. Myers R. J. Jr. Sánchez-Vicente M. I. Lorenzo O. Mittler R. (2025). Aboveground whole-plant live imaging method for nitric oxide (NO) reveals an intricate relationship between NO and H 2 O 2. New Phytologist. doi: 10.1111/nph.70094 40135542

[B58] Molassiotis A. Fotopoulos V. (2011). Oxidative and nitrosative signaling in plants: two branches in the same tree? Plant Signaling Behav. 6, 210–214. doi: 10.4161/psb.6.2.14878 PMC3121980 21325889

[B59] Nabati J. Nemati Z. Rezazadeh E. B. (2024). Involvement of nitric oxide in biochemical and physiological response of potato seedling under cold stress. J. Plant Growth Regul. 43, 4321–4332. doi: 10.1007/s00344-024-11401-z

[B60] Nabi R. B. S. Rolly N. K. Tayade R. Khan M. Shahid M. Yun B.-W. (2021). Enhanced resistance of atbzip62 against Pseudomonas syringae pv. tomato suggests negative regulation of plant basal defense and systemic acquired resistance by AtbZIP62 transcription factor. Int. J. Mol. Sci. 22, 11541. doi: 10.3390/ijms222111541 34768971 PMC8584143

[B61] Olin A. C. Alving K. Toren K. (2004). Exhaled nitric oxide: relation to sensitization and respiratory symptoms. Clin. Exp. Allergy 34, 221–226. doi: 10.1111/j.1365-2222.2004.01888.x 14987301

[B62] Ombale S. Bhatt M. Tiwari A. K. Sharma A. Tiwari B. S. (2025). Cellular nitro-oxidative burden and survival through regulated cell death in the plants. Protoplasma, 1–14. doi: 10.1007/s00709-025-02071-z 40325188

[B63] Ozdemir C. Isik B. Koca G. Inan M. A. (2024). Effects of mid−gestational sevoflurane and magnesium sulfate on maternal oxidative stress, inflammation and fetal brain histopathology. Exp. Ther. Med. 28, 286. doi: 10.3892/etm.2024.12574 38827470 PMC11140313

[B64] Paciência I. Cavaleiro Rufo J. Ribeiro A. Mendes F. Farraia M. Silva D.. (2021). Association between the density and type of trees around urban schools and exhaled nitric oxide levels in school children. Eur. Ann. Allergy Clin. Immunol. 53, 29–36. doi: 10.23822/EurAnnACI.1764-1489.162 32729315

[B65] Pande A. Mun B.-G. Lee D.-S. Khan M. Lee G.-M. Hussain A.. (2021). No network for plant–microbe communication underground: A review. Front. Plant Sci. 12, 658679. doi: 10.3389/fpls.2021.658679 33815456 PMC8010196

[B66] Parisi C. Pastore A. Stornaiuolo M. Sortino S. (2024). A fluorescent probe with an ultra-rapid response to nitric oxide. J. Materials Chem. B. 12, 5076–5084. doi: 10.1039/D4TB00064A 38567488

[B67] Paul P. Sharma S. Pandey R. (2023). Phosphorus scavenging and remobilization from root cell walls under combined nitrogen and phosphorus stress is regulated by phytohormones and nitric oxide cross-talk in wheat. J. Plant Growth Regul. 42, 1614–1630. doi: 10.1007/s00344-022-10646-w

[B68] Pavlopoulos G. A. Secrier M. Moschopoulos C. N. Soldatos T. G. Kossida S. Aerts J.. (2011). Using graph theory to analyze biological networks. BioData Min. 4, 1–27. doi: 10.1186/1756-0381-4-10 21527005 PMC3101653

[B69] Pinu F. R. Beale D. J. Paten A. M. Kouremenos K. Swarup S. Schirra H. J.. (2019). Systems biology and multi-omics integration: viewpoints from the metabolomics research community. Metabolites 9, 76. doi: 10.3390/metabo9040076 31003499 PMC6523452

[B70] Pires J. Sousa S. Pereira M. Alvim-Ferraz M. Martins F. (2008). Management of air quality monitoring using principal component and cluster analysis—Part II: CO, NO2 and O3. Atmospheric Environ. 42, 1261–1274. doi: 10.1016/j.atmosenv.2007.10.041

[B71] Pirooz P. Amooaghaie R. Ahadi A. Sharififar F. (2021). Silicon-induced nitric oxide burst modulates systemic defensive responses of Salvia officinalis under copper toxicity. Plant Physiol. Biochem. 162, 752–761. doi: 10.1016/j.plaphy.2021.02.048 33799186

[B72] Rizwan M. Mostofa M. G. Ahmad M. Z. Imtiaz M. Mehmood S. Adeel M.. (2018). Nitric oxide induces rice tolerance to excessive nickel by regulating nickel uptake, reactive oxygen species detoxification and defense-related gene expression. Chemosphere 191, 23–35. doi: 10.1016/j.chemosphere.2017.09.068 29028538

[B73] Rosseel Y. (2012). lavaan: An R package for structural equation modeling. J. Stat. software 48, 1–36. doi: 10.18637/jss.v048.i02

[B74] Ryan T. P. (2013). Sample size determination and power (Hoboken, NJ, USA: John Wiley & Sons).

[B75] Saini S. Sharma P. Singh P. Kumar V. Yadav P. Sharma A. (2023). Nitric oxide: An emerging warrior of plant physiology under abiotic stress. Nitric. Oxide 140, 58–76. doi: 10.1016/j.niox.2023.10.001 37848156

[B76] Shu P. Sheng J. Qing Y. Shen L. (2025). SlATG5 is crucial for the accumulation of ROS in postharvest tomato fruit resistance to B. cinerea mediated by nitric oxide. Postharvest Biol. Technol. 219, 113204. doi: 10.1016/j.postharvbio.2024.113204

[B77] Singh R. K. Chaudhary B. D. (1981). Biometrical methods in quantitative genetic analysis (New Delhi, India: Kalyani Publishers).

[B78] Singh R. Mangat N. S. Singh R. Mangat N. S. (1996). Stratified sampling. Elements survey sampling Vol. 15 (Dordrecht, Netherlands: Kluwer Academic Publishers). pp. 102–144. doi: 10.1007/978-94-017-1404-4

[B79] Singh K. S. van der Hooft J. J. van Wees S. C. Medema M. H. (2022). Integrative omics approaches for biosynthetic pathway discovery in plants. Natural Product Rep. 39, 1876–1896. doi: 10.1039/D2NP00032F PMC9491492 35997060

[B80] Song K. Li H. Yang K. Ma T. Hu Y. Chen J.. (2025). Exogenous sodium nitroprusside exhibits multiple positive roles in alleviating cadmium toxicity in tobacco (Nicotiana tabacum L.). Nitric. Oxide 154, 8–18. doi: 10.1016/j.niox.2024.11.002 39547540

[B81] Sparacino-Watkins C. E. Lancaster J. R. Jr. (2021). Direct measurement of nitric oxide (NO) production rates from enzymes using ozone-based gas-phase chemiluminescence (CL). Nitric. Oxide 117, 60–71. doi: 10.1016/j.niox.2021.10.001 34653611

[B82] Tahjib-Ul-Arif M. Wei X. Jahan I. Hasanuzzaman M. Sabuj Z. H. Zulfiqar F.. (2022). Exogenous nitric oxide promotes salinity tolerance in plants: A meta-analysis. Front. Plant Sci. 13, 957735. doi: 10.3389/fpls.2022.957735 36420041 PMC9676926

[B83] Tang S. Xie Y. Yuan C. Sun X. Cui Y. (2019). Fractional exhaled nitric oxide for the diagnosis of childhood asthma: a systematic review and meta-analysis. Clin. Rev. Allergy Immunol. 56, 129–138. doi: 10.1007/s12016-016-8573-4 27444490

[B84] Tian D. Chen Z. Lin Y. Chen Z. Bui K. T. Wang Z.. (2020). Weighted gene co-expression network coupled with a critical-time-point analysis during pathogenesis for predicting the molecular mechanism underlying blast resistance in rice. Rice 13, 1–14. doi: 10.1186/s12284-020-00439-8 33306159 PMC7732884

[B85] Vazquez A. (2011). “Protein interaction networks,” in Neuroproteomics ed. Alzate O. (Boca Raton, FL, USA: CRC Press/Taylor & Francis). 21882453

[B86] Wang Y. Cao Y. Li Y. Yuan M. Xu J. Li J. (2023). Identification of key signaling pathways and hub genes related to immune infiltration in Kawasaki disease with resistance to intravenous immunoglobulin based on weighted gene co-expression network analysis. Front. Mol. Biosci. 10, 1182512. doi: 10.3389/fmolb.2023.1182512 37325483 PMC10267737

[B87] Wang M. Zhao B. Jiang N. Li H. Cai J. (2024). Advancing nitrogen nutrition index estimation in summer maize using continuous wavelet transform. Front. Plant Sci. 15, 1478162. doi: 10.3389/fpls.2024.1478162 39588096 PMC11586213

[B88] Wink D. A. Hines H. B. Cheng R. Y. Switzer C. H. Flores-Santana W. Vitek M. P.. (2011). Nitric oxide and redox mechanisms in the immune response. J. leukocyte Biol. 89, 873–891. doi: 10.1189/jlb.1010550 21233414 PMC3100761

[B89] Wood D. (2015). Limit of detection (LOD) and limit of quantification (LOQ). Local Authority Waste Recycl 2015, 11–13.

[B90] Yoshioka H. Asai S. Yoshioka M. Kobayashi M. (2009). Molecular mechanisms of generation for nitric oxide and reactive oxygen species, and role of the radical burst in plant immunity. Molecules Cells 28, 321–330. doi: 10.1007/s10059-009-0156-2 19830396

[B91] Yu H. Le Roux J. J. Jiang Z. Sun F. Peng C. Li W. (2021). Soil nitrogen dynamics and competition during plant invasion: insights from Mikania micrantha invasions in China. New Phytol. 229, 3440–3452. doi: 10.1111/nph.17125 33259063

[B92] Zeiger R. S. Schatz M. Zhang F. Crawford W. W. Kaplan M. S. Roth R. M.. (2011). Elevated exhaled nitric oxide is a clinical indicator of future uncontrolled asthma in asthmatic patients on inhaled corticosteroids. J. Allergy Clin. Immunol. 128, 412–414. doi: 10.1016/j.jaci.2011.06.008 21807253

[B93] Zhang H.-X. Chen J.-B. Guo X.-F. Wang H. Zhang H.-S. (2014). Highly sensitive low-background fluorescent probes for imaging of nitric oxide in cells and tissues. Analytical Chem. 86, 3115–3123. doi: 10.1021/ac4041718 24564742

[B94] Zhang Z.-W. Fu Y.-F. Zhou Y.-H. Wang C.-Q. Lan T. Chen G.-D.. (2019). Nitrogen and nitric oxide regulate Arabidopsis flowering differently. Plant Sci. 284, 177–184. doi: 10.1016/j.plantsci.2019.04.015 31084870

[B95] Zhang Y. Wang R. Wang X. Zhao C. Shen H. Yang L. (2023). Nitric oxide regulates seed germination by integrating multiple signalling pathways. Int. J. Mol. Sci. 24, 9052. doi: 10.3390/ijms24109052 37240398 PMC10219172

[B96] Zhao Q.-P. Wang J. Yan H.-R. Yang M.-Y. Wang J. Zhao X.. (2021). Nitric Oxide Associated Protein1 (AtNOA1) is necessary for copper-induced lateral root elongation in Arabidopsis thaliana. Environ. Exp. Bot. 189, 104544. doi: 10.1016/j.envexpbot.2021.104544

[B97] Zhou X. Joshi S. Khare T. Patil S. Shang J. Kumar V. (2021). Nitric oxide, crosstalk with stress regulators and plant abiotic stress tolerance. Plant Cell Rep. 40, 1395–1414. doi: 10.1007/s00299-021-02705-5 33974111

